# Functional independence profile of critically ill patients

**DOI:** 10.1186/cc14680

**Published:** 2015-09-28

**Authors:** Karina Tavares Timenetsky, José AS Junior, Andréia SA Cancio, Angela SY Yang, Carolina SA Azevedo, Cilene SM Silva, Corinne Taniguchi, Daniela Nobrega, Fernanda Domingues, Juliana Raimondo, Louise HR Gonçalves, Pedro Veríssimo, Raquel AC Eid

**Affiliations:** 1 Hospital Israelita Albert Einstein, Morumbi, São Paulo, São Paulo, Brazil

## Introduction

The functional independence measure (FIM) is an outcome measure of the severity of physical and cognitive disability for an inpatient rehabilitation setting. The severity of disability changes during rehabilitation treatment, making changes in the FIM scale an indicator of treatment benefits and its results. So far there is no evidence for the functional profile of patients followed by physiotherapists during their critically ill department stay.

## Objective

This study objective was to evaluate the functional profile of critically ill patients during their hospital stay through the FIM scale.

## Methods

A cohort study evaluating the FIM scale of critically ill patients admitted to Hospital Israelita Albert Einstein, older than 18 years old, during a 1-year period (January-December 2014), that were followed by physiotherapists. The FIM scale was applied by trained and certified physiotherapists at day 1 (when the patient was alert and responsive), at ICU discharge and at critically ill department discharge. The FIM scale rates from 18 to 126 points, evaluating: self-care, sphincter control, transfer and mobility, deambulation, communication and social cognition. With the FIM scale, patients were characterized as independent, modified dependence, or complete dependence.

## Results

A total of 1457 patients were included in the study, mean age of 85 (±4.5) years. Of these patients, 56% were male. The most frequent admission diagnosis was respiratory disorders in 28.4% of the patients, followed by 18% cardiac reasons, 16.4% infection, and 11.5% neurological disorders. The median FIM scale was 62 points (48-81) at day 1 and 83 points (57-99) at critically ill department discharge. There was a significant improvement in FIM scale at the critically ill department discharge (*p *<0.001). Patients that were discharged from the ICU had a lower median FIM scale when compared with the step-down unit and coronary unit (80, 54-99 vs. 90, 71-99 vs. 54, 39-64 respectively; *p *<0.0001).

## Conclusion

The FIM scale improved significantly during the critically ill department stay for patients accompanied by physiotherapists. Although at ICU discharge patients have a lower FIM scale when compared with the step-down unit and coronary unit, probably due to the short length of stay.

